# Differential expression of cysteine desulfurases in soybean

**DOI:** 10.1186/1471-2229-11-166

**Published:** 2011-11-18

**Authors:** Marta D Heis, Elisabeth M Ditmer, Luisa A de Oliveira, Ana Paula G Frazzon, Rogério Margis, Jeverson Frazzon

**Affiliations:** 1 Biotechnology Center, Federal University of Rio Grande do Sul - UFRGS, Porto Alegre, RS, Brazil; 2 Department of Microbiology, Federal University of Rio Grande do Sul - UFRGS, Porto Alegre, RS, Brazil; 3 Department of Food Science, Federal University of Rio Grande do Sul - UFRGS, Porto Alegre, RS, Brazil

## Abstract

**Background:**

Iron-sulfur [Fe-S] clusters are prosthetic groups required to sustain fundamental life processes including electron transfer, metabolic reactions, sensing, signaling, gene regulation and stabilization of protein structures. In plants, the biogenesis of Fe-S protein is compartmentalized and adapted to specific needs of the cell. Many environmental factors affect plant development and limit productivity and geographical distribution. The impact of these limiting factors is particularly relevant for major crops, such as soybean, which has worldwide economic importance.

**Results:**

Here we analyze the transcriptional profile of the soybean cysteine desulfurases *NFS1*, *NFS2 *and *ISD11 *genes, involved in the biogenesis of [Fe-S] clusters, by quantitative RT-PCR. *NFS1*, *ISD11 *and *NFS2 *encoding two mitochondrial and one plastid located proteins, respectively, are duplicated and showed distinct transcript levels considering tissue and stress response. *NFS1 *and *ISD11 *are highly expressed in roots, whereas *NFS2 *showed no differential expression in tissues. Cold-treated plants showed a decrease in *NFS2 *and *ISD11 *transcript levels in roots, and an increased expression of *NFS1 *and *ISD11 *genes in leaves. Plants treated with salicylic acid exhibited increased *NFS1 *transcript levels in roots but lower levels in leaves. *In silico *analysis of promoter regions indicated the presence of different *cis*-elements in cysteine desulfurase genes, in good agreement with differential expression of each locus. Our data also showed that increasing of transcript levels of mitochondrial genes, *NFS1*/*ISD11*, are associated with higher activities of aldehyde oxidase and xanthine dehydrogenase, two cytosolic Fe-S proteins.

**Conclusions:**

Our results suggest a relationship between gene expression pattern, biochemical effects, and transcription factor binding sites in promoter regions of cysteine desulfurase genes. Moreover, data show proportionality between *NFS1 *and *ISD11 *genes expression.

## Background

[Fe-S] clusters may be the most ancient and versatile inorganic cofactors in biological systems. They can be found in all living organisms, participating in electron transfer, catalysis and regulatory processes. Besides, [Fe-S] clusters are involved in sensing environmental stimuli and regulation of protein expression [1-3]. In plants, the biogenesis of Fe-S proteins is compartmentalized and mostly adapted to the requirements of the green tissue, which carries out both photosynthesis and respiration, processes that require significant amounts of Fe-S proteins. Mitochondria and plastid have their own machineries for [Fe-S] cluster assembly, which differ in biochemical and genetic properties. Among the Fe-S proteins known in plant mitochondria are complexes I, II and III of the respiratory chain and aconitase of the citric acid cycle, and in plastids are cytochrome *b*_6_*f *complex, photosystem I and ferredoxin-thioredoxin reductase [4-7].

Three different systems for [Fe-S] clusters biosynthesis have been identified in bacteria, all of them share cysteine desulfurases and [Fe-S] cluster scaffold proteins. Those systems are referred to as NIF (nitrogen fixation system), ISC (iron-sulfur cluster assembly system) and SUF (sulfur mobilization system) [8-10]. There are several mitochondrial proteins homologous to the bacterial ISC system, including a group I NifS-like proteins, supporting the evolutionary relationship between α *Proteobacteria *and mitochondria [5]. In yeast, it has been shown that mitochondria are the primary site of [Fe-S] cluster formation; however, these organelles not only produce their own Fe-S proteins, but are also required for the maturation of cytosolic Fe-S proteins [11]. In the chloroplast, five different [Fe-S] cluster types are found in various proteins, and this organelle possesses its own machinery for [Fe-S] biosynthesis which is most similar to those found in cyanobacteria containing the SUF system and the cysteine desulfurase which is similar to the bacterial SufS, a group II NifS-like protein [12,13].

Cysteine desulfurase is a pyridoxal 5'-phosphate (PLP)-dependent enzyme that catalyzes the conversion of L-cysteine to L-alanine and sulfane sulfur. This occurs through the formation of a protein-bound cysteine persulfide intermediate on a conserved cysteine residue [14,15]. Considering that sulfide and free iron are toxic to the cell, intracellular concentrations are thought to be extremely low. Besides being involved in sulfur mobilization, cysteine desulfurase is proposed to be involved in cellular iron homeostasis [16-18]. ISD11 is an essential mitochondria matrix protein, a component of the ISC-assembly machinery, and is conserved in eukaryotes, but not found in prokaryotes. This protein forms a stable complex with NFS1, increases NFS1 activity, and is essential for the enzymatic activity of several Fe-S proteins [19,20]. Sulfur-containing defense compounds (SDCs) are involved in stress response and their synthesis involve several genes for sulfur assimilation [21].

It is hypothesized that soybean (*Glycine max*) has gone through at least two polyploidy and diploidization events, being considered a paleopolyploid [22], still presenting many gene duplications [23]. Various stresses can adversely affect plant growth and crop production, such as low temperature which modifies membrane lipid composition, thus affecting mitochondria respiratory function [24] and presumably photosynthesis. Expression of various plant genes is regulated by abiotic environmental stresses such as cold. Many *cis*-acting elements involved in stress response and stress-inducible genes contain *cis*-acting elements in their promoter regions have been described.

Here, we identified the soybean cysteine desulfurase genes by sequence comparison. Furthermore, we investigate the responsiveness of these genes under biotic and abiotic stresses, as well as transcript distribution in different tissues. Association between the high transcript level of mitochondrial genes, *NFS1 *and *ISD11*, and an increased expression of two cytosolic Fe-S proteins is showing here. Our data also demonstrate the relationship between the presence of specific *cis*-elements and regulation of transcript levels under various conditions.

## Results

### Sequence analysis

Comparative protein analyses showed that there are four cysteine desulfurase genes in *G. max*, corresponding to loci Glyma01g40510, Glyma09g02450, Glyma11g04800 and Glyma15g13350. These proteins can be classified into two groups: the first group is composed of IscS-like proteins, mitochondrial cysteine desulfurases, which are encoded by the genes located on chromosome 01 and 11 (*NFS1_Chr01 *and *NFS1_Chr11*); the second group encompasses SufS-like proteins, plastid cysteine desulfurases, which are encoded by genes located on chromosome 9 and chromosome 15 (*NFS2_Chr09 *and *NFS2_Chr15*). Soybean *NFS1 *genes share 94% nucleic acid similarity and 98% protein identity, while *NFS2 *genes share 96% nucleic acid similarity and 97% protein identity. When compared to *Arabidopsis thaliana *sequences, NFS1 proteins have 76% protein identity, whereas NFS2 have 77%. Pfam analysis demonstrated that all genes encode for an aminotransferase class-V motif and alignment analysis showed the location of a cysteine in the active site and a histidine and alanine in the cofactor binding site (Additional files 1 and 2). To find *ISD11 *genes, we used sequences from S*accharomyces cerevisiae *and *A. thaliana *as queries against the Glyma1 genome. We found two loci that encode ISD11 orthologs, Glyma08g26490 and Glyma18g49970, showing 87% protein identity (Additional file 3). Soybean genes that encode NFS1, NFS2 and ISD11 appear at least twice on different chromosomes due to duplication events [25].

### Phylogenetic analysis

**Figure 1:**
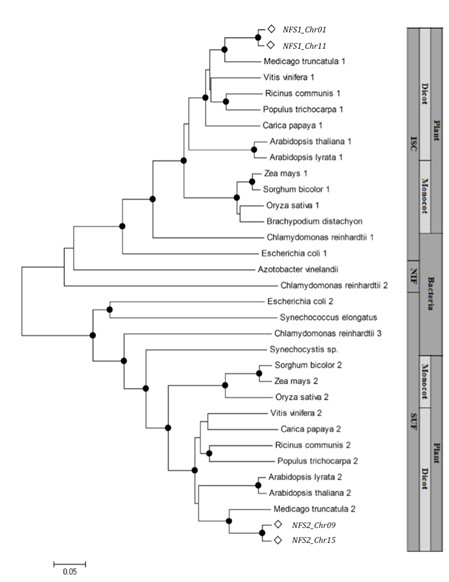
**Phylogenetic analysis of cysteine desulfurase proteins**. It is indicated to which [Fe-S] cluster biosynthesis systems (ISC, NIF and SUF) cysteine desulfurase belongs, and if this is a bacterial or plant (monocot or dicot) sequence. Black dots indicate bootstrap value higher than 80%.

Comparative amino acid analysis of IscS-like and SufS-like proteins of different plants and bacterial species showed that conserved regions varied from 54 to 98% and from 37 to 97% identity, respectively. A phylogenetic analysis of a wide range of organisms has shown that cysteine desulfurases form three independent clusters (Figure 1). One clade was composed by all sequences from the ISC system, and divided into monocots, dicots and bacteria, forming three subclades. The second cluster contained bacterial and algae sequences of cysteine desulfurases from the NIF system. The third clade was composed of proteins from the SUF system, and subdivided into three clades, showing the same branching as the ISC system. Some bacterial and all mitochondria located proteins clustered together; some bacterial, cyanobacterial and all plastid located proteins were also found in one cluster. This is in agreement with the endosymbiotic theory, which establishes a relationship between the endosymbiotic host and the bacterial ancestors [5].

### Transcript analysis of cysteine desulfurases and ISD11 genes in soybean

Considering that Fe-S proteins are involved in environmental or cellular sensing [5], quantitative RT-PCR was performed in order to investigate transcript levels of cysteine desulfurases and *ISD11 *in soybean. We designed gene-specific primers for *NFS1_Chr01*, *NFS1-_Chr11*, *NFS2_Chr09 *and *NFS2_Chr15 *and analyzed the expression pattern of leaves and roots from non-treated plants and plants treated with salicylic acid (SA) and cold incubation. Further, to study whether *ISD11 *transcript levels are co-regulated with the *NFS1 *expression pattern, we performed a quantitative RT-PCR with non-treated and cold-treated plants. For all studied genes the transcript levels were normalized to the transcript levels of *F-BOX *and *Metalloprotease *[26].

**Figure 2:**
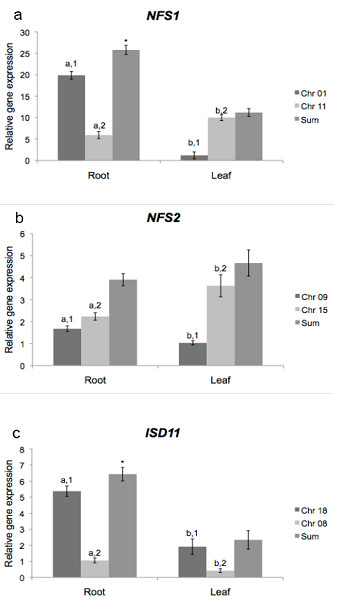
**NFS1, NFS2 and ISD11 gene expression in root and leaf**. Quantitative RT-PCR analysis of (a) *NFS1*, (b) *NFS2 *and (c) *ISD11 *gene expression in soybean tissues from total root and leaf RNA. Relative expression level was measured by performing PCR in four biological replicates and four technical replicates for each biological replicate per tissue with SE shown. Values were normalized against *F-BOX *and *MET*. *a *and *b *indicate difference between tissues for each gene. *1 *and *2 *indicate difference between genes in each tissue. * indicates difference in sum.

In order to determine whether duplicated genes have differential expression profiles, we analyzed mRNA accumulation in control plants. These analyses showed that each cysteine desulfurase gene is individually expressed indicating a differential response to environmental stimuli. As the duplicated genes share a high degree amino acid identity, we summed expression levels from both copies to compare total *NFS1 *and *NFS2 *mRNA accumulation. While *NFS1_Chr01 *is predominantly expressed in roots, we found higher *NFS1_Chr11 *transcript levels in leaves. In sum, a higher level is found in roots (Figure 2a). *NFS2_Chr15 *shows a higher expression in both organs as compared to *NFS2_Chr09*. *NFS2_Chr09 *transcripts mostly accumulate in roots and those of *NFS2_Chr15 *in leaves (Figure 2b). In both organs, *ISD11_Chr18 *shows a higher expression level than *ISD11_Chr08*. Taken together, a higher level was found in roots (Figure 2c). As shown in Genevestigator database, *NFS1 *is highly expressed in roots than in leaves in *Arabidopsis thaliana*, while *NFS2 *is predominantly expressed in leaves [27].

**Figure 3:**
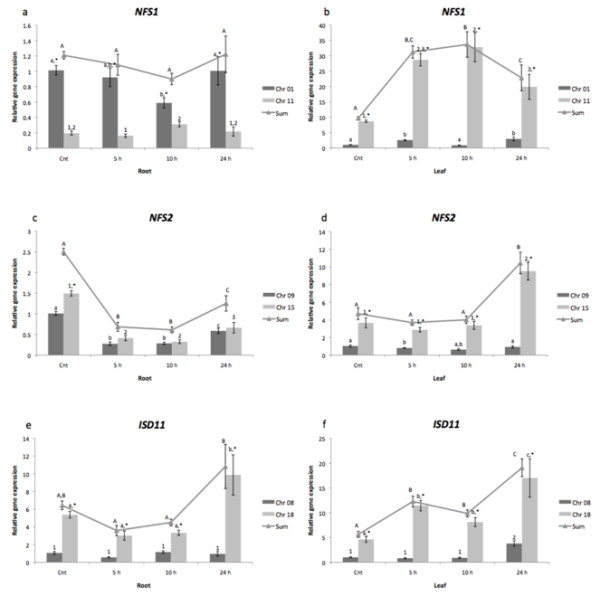
**NFS1, NFS2 and ISD11 gene expression in cold-treated plants**. Quantitative RT-PCR analysis of *NFS1 *gene expression in (a) root and (b) leaf, *NFS2 *gene expression in (c) root and (d) leaf and *ISD11 *gene expression in (e) root and (f) leaf from cold-treated plants. Relative expression level was measured by performing qPCR in four biological replicates and four technical replicates for each biological replicate per tissue with SE shown. Values were normalized against *F-BOX *and *MET*. *Letters *or *numbers *indicate difference in transcription level among time-points analyzed. * indicates difference in transcription level between duplicated genes at one point.

It appeared that cold-treated plants exhibited a differential response depending on the gene and tissue. In roots, *NFS1_Chr01 *showed a higher expression than *NFS1_Chr11 *during the whole treatment. While *NFS1_Chr01 *transcript level decreased upon cold treatment, those of *NFS1_Chr11 *increased (Figure 3a). Sum analysis showed that total *NFS1 *mRNA in roots did not respond to cold treatment (Figure 3). In leaves, *NFS1_Chr11 *was higher expressed than *NFS1_Chr01 *during the whole treatment. *NFS1_Chr01 *transcript level oscillated, and *NFS1_Chr11 *increased its expression during cold treatment (Figure 3b). Total *NFS1 *mRNA increased in leaves after cold incubation (Figure 3). These results corroborate with *A. thaliana *database, where leaves improve expression during cold treatment, while roots do not change [27]. Cold-treatment induced a decrease in both *NFS2_Chr09 *and *NFS2_Chr15 *transcript levels in roots reaching a comparable expression level at 5, 10 and 24 h (Figure 3c) (Figure 3). In leaves, *NFS2_Chr15 *showed a higher expression level than *NFS2_Chr09 *during the whole treatment. While *NFS2_Chr09 *transcript levels decreased, *NFS2_Chr15 *increased at 24 h (Figure 3d). Sum analysis showed that cold-treatment induced a decrease in *NFS2 *genes transcript levels (Figure 3). In roots, *ISD11_Chr18 *was higher expressed than *ISD11_Chr08 *during the whole treatment. While *ISD11_Chr18 *transcript levels increased were cold induced, *ISD11_Chr08 *did not show changes in expression (Figure 3e). Sum analysis revealed that total *ISD11 *mRNA initially decreased and then reached the former level in roots upon cold treatment (Figure 3). In leaves, *ISD11_Chr18 *showed a higher expression than *ISD11_Chr08 *during whole treatment, but both genes were upregulated upon cold treatment (Figure 3f).

**Figure 4:**
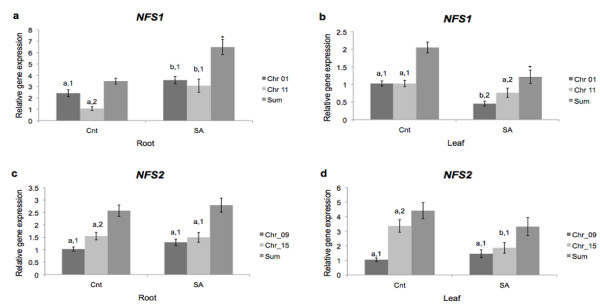
**NFS1 and NFS2 gene expression in SA-treated plants**. Quantitative RT-PCR analysis of *NFS1 *gene expression in (a) root and (b) leaf, and *NFS2 *gene expression in (c) root and (d) leaf from SA-treated plants. Relative expression level was measured by performing qPCR in four biological replicates and four technical replicates for each biological replicate per tissue with SE shown. Values were normalized against *F-BOX *and *MET*. *a *and *b *indicate difference between tissues for each gene. *1 *and *2 *indicate difference between genes in each tissue. * indicates difference in sum.

When treated with 2 mM SA, the response of cysteine desulfurase expression varies depending on tissue and gene. In roots, both *NFS1 *genes were upregulated upon SA incubation. Thus, sum analysis showed that *NFS1 *mRNA level after SA treatment was significantly higher than before (Figure 4a). In leaves, *NFS1_Chr01 *decreased expression, while *NFS1_Chr11 *did not show any changes. Total *NFS1 *mRNA level decreased due to SA treatment (Figure 4b). When *A. thaliana *were treated with 2 mM SA during 24 hours, *NFS1 *expression was induced [27]. *NFS2 *transcript levels did not change in roots (Figure 4c). In leaves, *NFS2_Chr15 *showed a significant decrease in expression, while *NFS2_Chr09 *did not change. Sum analysis, for leaf, did not show any changes in expression (Figure 4d).

### Cis-elements search in promoter regions

**Figure 5:**
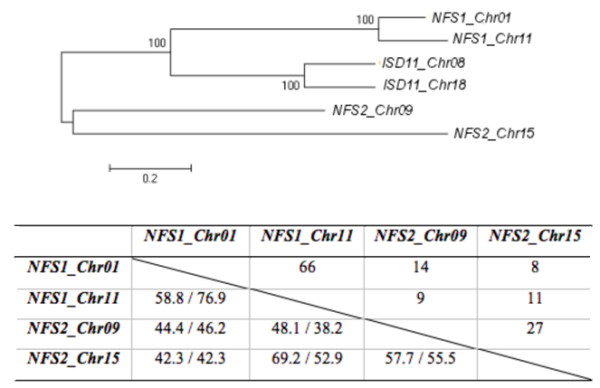
**Phylogenetic analysis of promoter regions**. Phylogenetic analysis of promoter regions of soybean *NFS1*, *NFS2 *and *ISD11 *genes. Numbers at branches indicate percentage of bootstrap values from 1,000 trials. As shown in the table below, sequence identity (%) between 1,500 bp upstream regions from transcriptional start site of soybean genes (top triangle) and percentage of common motifs between genes (bottom triangle).

To identify putative *cis*-elements present in the *NFS1 *and *NFS2 *promoters, we inspected the sequences 1,500 bp upstream of the transcriptional start site of all genes using the Plant *Cis*-Acting Regulatory Elements (PlantCARE) database [28]. The analysis identified a total of 178, 168, 163 and 151 hits for potential *cis*-elements putative transcription factor binding sites in *NFS1_Chr01*, *NFS1_Chr11*, *NFS2_Chr09 *and *NFS2_Chr15*, respectively. While some of the predicted *cis*-elements were present multiple times in the promoters, others occurred only once. All putative transcription factor binding sites with known function are shown in Table 1. Comparative analysis among cysteine desulfurase promoter regions showed sequence similarity between 8 and 66%, and that the amount of shared *cis*-elements varies from 38.2 to 76.9% (Figure 5). Comparing duplicated genes, they have a high promoter region similarity, and *NFS1 *and *ISD11 *promoters diverged less than those of *NFS2 *genes (Figure 5). The relationship between some motifs and our quantitative RT-PCR results are shown in Table 2.

**Table 1:** Transcription factor binding sites and motifs.

Motifs	*NFS1* *Chr01*	*NFS1* *Chr11*	*NFS2* *Chr09*	*NFS2* *Chr15*	Function
3-AF1 binding site			x		light responsive element
**5UTR Py-rich stretch**		**x (1)**	**x (1)**	**x (2)**	***cis*-acting element conferring high transcription level**
ABRE				x	*cis*-acting element involved in the abs*cis*ic acid responsiveness
ACE	x	x			*cis*-acting element involved in light responsiveness
AE-box	x				part of a module for light response
**ARE**	**x (1)**		**x (1)**	**x (1)**	***cis*-acting regulatory element essential for anaerobic induction**
**as-2-box**		**x (1)**		**x (1)**	**involved in shoot-specific expression and light responsiveness**
AT- rich element		x			binding site of AT-rich DNA binding protein (ATBP-1)
AT1-motif				x	part of a light responsive module
ATCT-motif	x				part of a conserved DNA module involved in light responsiveness
Box 4	x	x	x	x	part of a conserved DNA module involved in light responsiveness
Box I	x	x	x	x	light responsive element
Box II			x		part of a light responsive element
Box III	x				protein binding site
Box W1			x	x	fungal elicitor responsive element
CAAT-box	x	x	x	x	common *cis*-element in promoter and enhancer regions
CAT-box			x	x	*cis*-acting regulatory element related to meristem expression
CATT-motif		x		x	part of a light responsive element
CCAAT-box			x	x	MYBHV1 binding site
CGTCA-motif	x	x			*cis*-acting regulatory element involved in MeJA-responsiveness
chs-CMA2a	x				part of a light responsive element
circadian	x	x	x		*cis*-acting regulatory element involved in circadian control
ERE		x			ethylene-responsive element
GAG-motif	x	x			part of a light responsive element
GA-motif	x	x	x		part of a light responsive element
GARE-motif		x		x	gibberillin-responsive element
G-Box			x	x	*cis*-acting regulatory element involved in light responsiveness
G-box			x	x	*cis*-acting regulatory element involved in light responsiveness
GT1-motif	x				light responsive element
HSE				x	*cis*-element involved in heat stress responsiveness
LAMP-element		x			part of a light responsive element
LS7				x	part of a light responsive element
**MBS**	**x (2)**	**x (2)**	**x (1)**	**x (1)**	**MYB binding site involved in drought-inducibility**
MBSI		x			MYB binding site involved in flavonoid biosynthetic genes regulation
MBSII		x			MYB binding site involved in flavonoid biosynthetic genes regulation
motif 1				x	*cis*-acting regulatory element root specific
MRE	x	x			MYB binding site involved in light responsiveness
P-box				x	gibberillin-responsive element
sdOCT	x				*cis*-acting regulatory element related to meristem specific activation
Skn-1 motif	x	x	x	x	*cis*-acting regulatory element required for endosperm expression
Sp1		x	x	x	light responsive element
TATA-box	x	x	x	x	core promoter element around -30 of transcription start
**TCA- element**	**x (1)**	**x (2)**	**x (2)**		***cis*-acting element involved in salicylic acid responsiveness**
**TC-rich repeats**	**x (2)**	**x (3)**	**x (2)**	**x (2)**	***cis*-acting responsive element involved in defense and stress responsiveness**
TCT-motif		x	x	x	part of a light responsive element
TGACG-motif	x	x			*cis*-acting regulatory element involved in MeJA-responsiveness
TGA-element	x	x			auxin-responsive element
**Total**	**23**	**27**	**21**	**25**	

Transcription factor binding sites and number of motifs in each 1.500 bp upstream regions from transcription start site of soybean genes, according to PlantCARE database in default parameters.

* The motifs cited in Table 2 are marked in bold and the number inside parenthesis represent the time it appeared.

**Table 2:** Relationship between motifs and qPCR.

*Cis*-element and organism^a^	Function	Gene	Correlation
5UTR Py-rich stretch *Lycopersicon esculentum*	*cis*-acting element conferring high transcription levels	*NFS1_Chr11*, *NFS2_Chr15*	*NFS2_Chr15 *is highly expressed in leaves and roots. *NFS1_Chr11 *is highly expressed in leaves.
ARE *Zea mays*	*cis*-acting regulatory element essential for the anaerobic induction	*NFS1_Chr01*	It is highly expressed in roots, where the O_2 _availability is low.
MBS *Arabidopsis thaliana*	MYB binding site involved in drought-inducibility	All	Drought stress effects are related to cold stress effects. All genes respond to cold.
TC-rich repeats *Nicotiana tabacum*	*cis*-acting element involved in defense and stress responsiveness	All	All genes respond to cold.
TCA-element *Brassica oleracea*	*cis*-acting element involved in salicylic acid responsiveness	*NFS1 *genes	Sum analysis showed that transcript level of *NFS1 *genes vary in SA treatment.
as-2-box *Nicotiana tabacum*	involved in shoot-specific expression and light responsiveness	*NFS1_Chr11*, *NFS2_Chr15*	Both genes are highly expressed in leaves.

Putative transcription factor binding sites within the *NFS1 *and *NFS2 *promoters that showed correlation to our qPCR data.

^a ^Organism where the *cis*-element was described, according to PlantCARE.

### Coincidence of increased NFS1/ISD11 transcript levels and activities of cytosolic Fe-S enzymes

**Figure 6:**
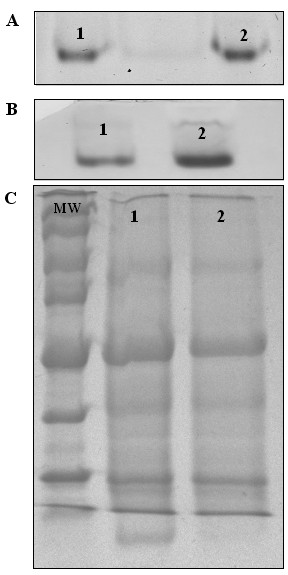
**Cold stress effects on AO and XDH activity**. (a) AO activity visualized by *in situ *staining after exposition of plants to cold stress for 18 h. Wells were loaded with 100 μg of protein of soybean wild type crude extracts of leaves from either untreated (1) or cold-stressed plants (2). Indole-3-carboxaldehyde plus 1-naphthaldehyde were used as substrate. (b) XDH activity visualized by *in situ *staining after exposition of plants to cold stress for 18 h. Wells were loaded with 100 μg of protein of soybean wild type crude extracts of leaves from either untreated (1) or cold-stressed plants (2). Hypoxanthine was used as substrate. (c) SDS PAGE gel 12% staining with Comassie blue. MW; molecular weight (Broad Range Protein Molecular Marked from Promega); wells were loaded with 100 μg of protein of soybean wild type crude extracts of leaves from either untreated (1) or cold-stressed plants (2).

Aldehyde oxidase (AO) catalyzes the conversion of an aldehyde to an acid and hydrogen peroxide in the presence of oxygen and water and Xanthine dehydrogenase (XDH) catalyzes the hydrogenation of xanthine to urate. Both enzymes require FAD, molybdenum and two [2Fe-2S] clusters as cofactors. Therefore, AO and XDH activities are directly dependent on the mitochondrial [Fe-S] cluster assembly machinery. Hence, we analyzed the activity of AO and XDH using an in-gel activity assay [29]. In comparison to unstressed leaves, XDH activities were clearly enhanced upon cold treatment while AO activities increased only moderately under these conditions. Crude extract were obtained from three independent treatment (Figure 6), indicating that *NFS1*/*ISD11 *are required for [Fe-S] cluster assembly on both proteins tested.

## Discussion

Soybean is a paleopolypoid plant, whose polyploidisation may have occurred in the common ancestor of the soybean and *Medicago truncatula*. In addition, it was suggested that a relatively recent polyploidy event occurred in the soybean lineage [22,30]. All analyzed genes are present in duplicate on different chromosomes showing a high degree of conservation and share important characteristics [25]. Due to the polyploidy events, mutations and gene rearrangements occurred, resulting in diversification of gene expression [31]. Here, we present the characterization of the promoters of soybean *NFS1*and *NFS2 *genes, and identified tissue- and stress-specific response in expression of cysteine desulfurase and *ISD11 *genes both involved in [Fe-S] cluster biosynthesis.

Three different systems responsible for [Fe-S] cluster biosynthesis have been described [3], and involved genes appear to be conserved in bacteria, fungi, animals and plants [6,32]. In our phylogenetic analysis (Figure 1), it was possible to identify three distinct groups, composed of proteins from ISC, NIF and SUF systems. *G. max *protein sequences were located in plant clades near to *M. truncatula*. Comparing our phylogenetic approach and the described polyploidy events [30], it was possible to hypothesize that analyzed cysteine desulfurase genes were duplicated after the divergence of soybean and *M. truncatula*. Thus, soybean has two copies of each cysteine desulfurase gene, while *M. truncatula *has only one copy (Figure 1). Both species contain duplicated *ISD11 *genes (data not shown). Therefore, this polyploidy event may have occurred prior to divergence of both lines.

The present results suggest that *NFS1 *and *NFS2 *soybean genes, which encode proteins involved in sulfur assimilation and [Fe-S] cluster biosynthesis [16], are involved in response to cold stress and SA. Sulfur is an essential macronutrient which is assimilated to cysteine [33,34], which will take part in the assembly of SDCs. When exposed to biotic and/or abiotic stress, synthesis of SDCs is induced via different signals, demonstrating their potential involvement in stress defense. There is an increased demand for cysteine as a precursor due to SDCs synthesis; therefore, the expression of genes for sulfur assimilation is induced [21]. Analyzing cold-treated plants, it is possible to observe that, in leaves, *NFS1 *and *NFS2 *genes increased transcript levels (Figure 3), perhaps due to SDCs stress response or due to its possible role in SDCs synthesis. When treated with SA, a simulator of biotic stress, *NFS1 *genes changed their expression pattern (Figure 4). In both experiments we observed a particular expression pattern, *i.e. *organs with primary contact to the stressor showed an increase in cysteine desulfurase transcript levels, while those less exposed showed a lower expression (Figures 3 and 4). This opposite profile may be due to a compensatory mechanism present in early stress response, and it may change if plants are exposed to longer stress periods.

When the plant cell are exposed to biotic or abiotic stress factors, modifications of the lipid composition of its membranes occur [35]. Soybean mitochondria show modifications in lipid content in response to low temperature [36], and this can alter respiratory properties and gene expression [24,37]. As many proteins involved in respiration, such as complexes I, II and III, are Fe-S proteins [4], a modification in the respiratory profile may change the requirement for proteins of the [Fe-S] cluster biosynthesis pathway, i.e. altering the expression of cysteine desulfurase genes. Stress dependent changes in gene expression occur in the cytoplasm as well as in chloroplasts. Whereas mitochondria developed an export system for [Fe-S] clusters that is essential for maturation of many nuclear and cytosolic proteins, [Fe-S] cluster biosynthesis in mitochondria has a direct impact on protein activity, such as for aldehyde oxidase and xanthine dehydrogenase [16,38,39] as shown in Figure 6. The chloroplast is extremely sensitive to abiotic stress factors, such as elevated temperature and light, both increasing reactive oxygen species. Glutathione is involved in protection against oxidative damage triggered by biotic and abiotic stress in the cytosol and other cellular compartments. Synthesis of this peptide depends on sulfur assimilation and cysteine synthesis [21,33,34], as this amino acid is the substrate of cysteine desulfurase [14,40], a change in cysteine content may lead to a modification in its catalytic properties.

SA and its methylated form are involved in development, and are also fundamental for hypersensitive response and for systemic acquired resistance under biotic stress [41,42]. SA can induce the formation of reactive oxygen species, and these can react with various molecules in the cell, including lipids. As the organelle is often exposed to strong oxidative stress, some antioxidant enzymes should be simultaneously upregulated. An alternative oxidase has been proposed to represent a functional marker for mitochondrial dysfunction during biotic stress, and its content is increased in SA-treated soybean [35,43]. The treatment with SA causes mitochondrial dysfunction via oxidative stress causing changes in the cysteine desulfurase expression. This enzyme transfers electrons from reduced ubiquinone to molecular oxygen, bypassing complexes III and IV [24], and complex III contains [Fe-S] cluster [4]. In addition, SA-treated soybean altered the fatty acid composition of its mitochondria. As these organelles modified their membranes upon SA treatment, and cellular respiration involves Fe-S proteins, the expression of cysteine desulfurase may be altered under biotic stress.

The present quantitative RT-PCR results revealed a relationship between *NFS1 *and *ISD11 *transcript contents in roots and leaves as both genes showed a similar expression pattern (Figure 2). Moreover, to analyze whether an increase in *NFS1 *expression triggers an increase in *ISD11 *transcript levels, we studied *ISD11 *expression levels under cold stress. In roots, total *ISD11 *mRNA decreased during the treatment and recovered to the initial level after 24 h, while *NFS1 *transcript levels did not change. In leaves, both genes were upregulated (Figure 3). The similarity in expression pattern between these genes may be explained by their function. NFS1 is a cysteine desulfurase involved in [Fe-S] biosynthesis in mitochondria [6], whereas Isd11 was recently identified in yeast as a protein responsible for forming a stable complex with Nfs1 [19,20]. Besides interacting with the cysteine desulfurase, ISD11 showed in humans an important role in mitochondrial and cytosolic iron homeostasis [44] mediated by NFS1 [18]. Here, we demonstrated that interaction between mitochondrial genes *NFS1/ISD11 *increased expression and maturation of cytosolic enzymes XDH and AO. These results corroborate with data described for yeast, which associates the mitochondrial machinery for [Fe-S] cluster biosynthesis as being responsible for maturation of cytosolic Fe-S proteins. Also, results in Figure 6, show, for the first time, a direct relation among increase expression of both cytosolic Fe-S protein (XDH and AO) and mitochondrial cysteine desulfurase as a result of cold stress conditions. Moreover, our results are in agreement with the experiments involved co-expression of NFS1 and ISD11 of *A. thaliana*, which show a higher stability of NFS1 when co-expressed with ISD11 may suggesting that the interaction of NFS1/ISD11 promote the correct conformational structure of NFS1 (de Oliveira, LA and Frazzon, APG personal communication).

The soybean genome contains highly similar genes integrated in wider regulatory networks involved in differential regulation, including the presence of *cis*-acting regulatory elements in promoter regions [31]. Therefore, we analyzed DNA sequences to predict putative transcription factor binding sites located in the -1500 bp promoter regions. Duplicated genes have highly homologous promoter regions (Figure 5). When *cis*-elements were compared, all genes share high degree of common binding sites (Figure 5), suggesting that cysteine desulfurase genes share regulatory networks. In spite of this similarity, it has been shown that different environmental factors may trigger gene expression (Figures 2, 3 and 4). Since a complex molecular network is involved in regulation of gene expression and transcription factors are important components that lead to activation or repression of transcription [45], the differences observed may be due to the requirements of the corresponding factors in a particular tissue or organelle. The analysis of transcription factor binding sites provided an insight into transcript level data. *Cis*-elements related to quantitative RT-PCR experiments are shown in Table 2. A *Py-rich *element was found in *NFS1_Chr11 *and *NFS2_Chr15 *genes that showed high transcription levels in leaves and in both leaves and roots, respectively. An *ARE *element was found in *NFS1_Chr01*, which displayed higher expression in roots than in leaves, whereas promoters with an *as-2-box *element showed higher expression in leaves. The *TCA-element*, related to SA response, was found in *NFS1 *genes that changed transcription pattern under this stress. Besides, all genes had *cis*-elements related to defense and stress (*TC-rich*) and to drought response (*MBS*), and several genes are induced by both drought and cold stress, indicating a crosstalk between signaling pathways [46].

## Conclusions

**Figure 7:**
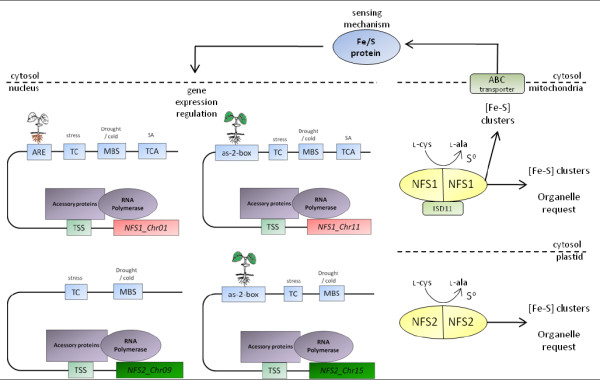
**Overview of expression control of NFS1 and NFS2 genes**. This model shows some *cis*-elements found within the promoter regions of cysteine desulfurases genes (green and red boxes). According to our qPCR data and *in silico *analysis those genes have particular expression triggers, indicated above the *cis*-elements (blue boxes). *NFS1_Chr01 *is modulated by stresses, such as cold and salicylic acid (SA), and induced in roots. *NFS1_Chr11 *is modulated by the same stimuli but induced in leaves. *NFS2_Chr09 *is modulated by cold, whereas *NFS2_Chr15 *is modulated by the same stress and induced in leaves. In plastid (bottom), cysteine desulfurase catalyses the release of sulfur from cysteine to [Fe-S] cluster biogenesis in order to organelle request. In mitochondria (top), the same process occurs to provide clusters to the organelle and cytosol/nucleus, being stimulated by a small protein called ISD11 (light green box). An export system, including an ABC transporter, is involved in maturation of Fe-S proteins outside mitochondria. Some Fe-S proteins in cytosol and nucleus are involved in cellular sensing, controlling gene expression.

In this study, we carried out an analysis of cysteine desulfurase genes from soybean, which are involved in [Fe-S] cluster biosynthesis. This study suggests that *NFS1 *and *NFS2 *genes are involved in stress response, and that their differential expression may be due to the presence of different *cis*-elements (Figure 7). Furthermore, *ISD11 *displayed an expression pattern similar to *NFS1 *genes, supporting a positive correlation in their activity. Our results provide the first insight into differential expression of duplicated genes involved in [Fe-S] cluster pathway, but further research is needed to determine whether other genes involved in [Fe-S] cluster biogenesis follow this pattern.

## Methods

### Identification of [Fe-S] cluster genes in soybean

To identify cysteine desulfurase-encoding genes from the annotations of Glyma1 in the soybean genome, a similarity search method was performed. We used a protein sequence data set of known cysteine desulfurases from *A. thaliana, Synechocystis *sp. and *Escherichia coli*, and the modeled proteome data of annotated genes downloaded from Phytozome [47]. To confirm the protein identity, sequences were subjected to a profile search using Pfam [48]. Besides, the search results for each cysteine desulfurase were then applied to retrieve discovered regions as conserved active sites and cofactor binding amino acids. Other genes that encode proteins involved in [Fe-S] cluster biosynthesis, such as *ISD11*, were found using the strategy described above.

### Phylogenetic analysis

Sequence alignments of cysteine desulfurase proteins were performed using ClustalX2 [49] with default parameters and then visualized with GeneDoc Program [50]. Phylogenetic analysis was made with the Molecular Evolutionary Genetic Analysis (MEGA) Package Version 4.0 [51] with Neighbor-Joining method. Pairwise deletion was used to analyze gap and missing data in the alignments. The bootstrap test was performed with 1000 replications, and only the bootstraps values higher than 80% are displayed as black dots on the nodes. The species and their respective access numbers in Phytozome or NCBI database are as follows: *G. max *(Glyma01g40510/Glyma11g04800/Glyma09g02450/Glyma15g13350), *Ricinus communis *(XP_002531989/XP_002523229), *A. thaliana *(At5g65720/At1g08490), *Zea mays *(ACF83040/NP_001130656), *Oryza sativa *(NP_001062914/EEC69110), *Chlamydomonas reinhardtii *(XP_001695008/XP_001701051/Au9.Cre12.g525650), *Brachypodium distachyon *(Bradi4g28300), *Arabidopsis lyrata *(XP_002864976/XP_002892460), *Sorghum bicolor *(Sb02g022360/Sb05g001270), *Carica papaya *(supercontig_129.49/supercontig_34.204), *Vitis vinifera *(XP_002274517/XP_002267920), *M. truncatula *(Medtr5g014960/Medtr3g105250), *Populus trichocarpa *(XP_002310363/XP_002314018), *E. coli *(YP_001458463/NP_289087), *Azotobacter vinelandii *(YP_002797401), *Synechococcus elongates *(NP_665776) and *Synechocystis *sp. (NP_442475).

### DNA sequence analysis

To predict the transcription factor binding sites located in the -1500 bp promoter regions of each soybean analyzed gene, we retrieved the -1500 bp upstream sequence from the putative transcription start site for each gene from the Glyma1 annotation. After that, the analysis of each promoter region was performed using the Plant *Cis*-Acting Regulatory Elements (PlantCARE) database [28]. Phylogenetic analysis of promoter regions was performed as described above, and percentage of bootstrap values are shown at branches.

### Plant material and treatments

To study the responsiveness of cysteine desulfurase genes under stress, two different experiments were performed. For cold and SA treatments, we used the soybean (*G. max *[L.] Merr.) cv. IAS-5 and cv. Conquista seeds, respectively. Plants were grown in plastic pots (3 seeds per pot), filled with vermiculite and solution of half-strength MS medium [52], in a controlled environment growth chamber at 16/8 h of day/night (22.5 μmol m^-2 ^s^-1^) photoperiod at 28 ± 1°C. For cold treatment, after 11 days, seedlings were transferred to a plastic pot, under the same conditions, containing one seedling. At the age of 27 days, plants were submitted to cold stress at 4°C for 0, 5, 10 and 24 hours. For SA treatment, plants were kept for 14 days in vermiculite, and then transferred to a hydroponic solution (half-strength MS medium) with additional 2 mM SA for 48 h. Control plants were submitted to the same conditions without SA addition. After both treatments, root and leaf tissues were collected, frozen in liquid nitrogen and stored at -80°C till further use. All the analyses were performed in biological quadruplicates.

### RNA isolation and quantitative RT-PCR

To study the expression of cysteine desulfurase genes, samples from control and treated plants were collected (*n *= 4 per each group). Total RNA was isolated from frozen tissues by extraction with Trizol (Invitrogen) according to the manufacturer's instructions and quality was evaluated by electrophoresis on a 1% agarose gel. Prior to quantitative RT-PCR, RNA samples were treated with *DNase *I (Promega) at 37°C for 30 min. Reverse transcription reactions were performed using the M-MLV reverse transcriptase (Invitrogen) following manufacturer's instructions.

Quantitative RT-PCR was conducted in an ABI 7500 Real-Time PCR System (Applied Biosystem) using SYBR Green I (Invitrogen) to detect double-strand cDNA synthesis. Soybean *F-BOX *(F-Box protein family) and *MET *(insulin-degrading enzyme, metalloprotease) genes were used as reference genes for data normalization and to calculate the relative mRNA levels. Reactions were done in a volume of 20 μL containing 10 μL of cDNA, 0.1 × SYBR Green I (Invitrogen), 0.025 mM dNTP, 1 × PCR Buffer, 3 mM MgCl_2_, 0.25 U Platinum Taq DNA Polimerase (Invitrogen), and 200 nM of each reverse and forward primers. A negative control without cDNA template was included for each primer combination. Primer sequences for quantitative RT-PCR were as follows: for *NFS1_Chr01*, NFS1_01R 5'-CCTCCCAATTCTCTCCATCGGT-3', and for *NFS1_Chr11*, NFS1_11R 5'-CCTCCCAATTTCCTCCATGGGC-3' with the same forward primer NFS1F 5'-CGGAGCACAAGTGCGTCC-3'; for *NFS2_Chr09*, NFS2_09R 5'-CCCGTGCACTTGAGCTGACA-3', and for *NFS2_Chr15*, NFS2_15R 5'-CACGTGCACTTGAGCTGACG-3' with the same forward primer NFS2F 5'-GTCGAACGAGCTGCCCTTTG-3'; for *ISD11_Chr08*, ISD11_08R 5'- CGCTGCGGAGCGGAGAAT-3', and for *ISD11_Chr18*, ISD11_18R 5'- TTGCGGAGCGGAGGGG -3' with the same forward primer ISD11F 5'-TCCACCGCCTTCGCCC-3'. These primers set generated amplicon sizes of 200, 157 and 91 and 89 bp for *NFS1*, *NFS2 *and ISD11, respectively (Additional file 4). The conditions were set as follows: an initial step for polymerase activation for 5 min at 94°C, 40 cycles of 15 s at 94°C for denaturation, 10 s at 60°C for annealing, and 15 s at 72°C for elongation, followed by a melting-curve analysis and fluorescence measured from 60 to 95°C. Four biological replicates and four technical replicates for each biological replicate were used.

### Activity assays

Total proteins extract from untreated and cold stress treatment tissue were obtained from plants grown in the same conditions as described for quantitative RT-PCR. Protein quantification was performed by Bradford assay (BioRad) and equal amount (100 μg) of protein was applied in a 7.5% native PAGE gel, AO and XDH were detected by activity staining previously described [29,53]

### Data analysis

Threshold and baselines were manually determined using the ABI 7500 Real-Time PCR SDS Software v2.0. To analyze the relative cysteine desulfurases mRNA expression relative to the constitutive genes, we used the 2^-ΔΔCt ^method [54]. Student's *t *test was performed to compare pairwise differences in gene expression, following the two samples assuming unequal variances and two-tailed distribution parameters. For time-course treatment, 1-way ANOVA and Duncan post hoc analysis were performed using SPSS17. The means were considered significantly different when *P *< 0.05.

## Authors' contributions

MDH carried out the quantitative RT-PCR analysis, sequence alignment, phylogenetic studies, *cis*-elements search, and performed the statistical analysis and drafted the manuscript. EMD performed the AO and XDS assays. LAO grow the plants and performed the RNA extraction and preparation of cDNA. APGF participated in the design of the study and coordination. RM performed the statistical analysis, participated in the design of the study and coordination. JF participated in the design and coordination of the study and gave the final approved. All authors read and approved the final manuscript.

## Supplementary Material

Additional file 1 **Alignment of IscS-like**. Alignment of soybean cysteine desulfurase homologue to IscS from *Escherichia coli*. * indicates residues from active and from cofactor binding sites. # indicates amino acids residues that differ between soybean duplicated genes. Click here for file

Additional file 2 **Alignment of SufS-like**. Alignment of soybean cysteine desulfurase homologue to SufS from *Escherichia coli*. * indicates residues from active and from cofactor binding sites. # indicates amino acids residues that differ between soybean duplicated genes. Click here for file

Additional file 3 **Alignment of ISD11 proteins**. Alignment of soybean, *Arabidopsis thaliana *and S*accharomyces cerevisiae *ISD11 proteins. Click here for file

Additional file 4 **Primer sequences**. Primer sequences and amplicon characteristics for each gene. Click here for file
