# Supplementary material for: Differential expression of cysteine desulfurases in soybean
Source: BMC Plant Biol. 2011 Nov 18;11:166. doi: 10.1186/1471-2229-11-166 (PMC3233524; doi:10.1186/1471-2229-11-166)
Supplement: Additional file 1 — Alignment of IscS-like. Alignment of soybean cysteine desulfurase homologue to IscS from Escherichia coli. * indicates residues from active and from cofactor binding sites. # indicates amino acids residues that differ between soybean duplicated genes. [file 1471-2229-11-166-S1.PDF]

```
                                                       #
Glycine_max_Chr01    : MTSKLIASNLRHQITKSAQNLRR-QLLSTAAAAAVPEHDGNNSNSLGMTMKGVRISGRPLYLDVQATSPVDPRVL :  74
Glycine_max_Chr11    : MTSKLIASNLRHQITKSAQNLRR-QLLSTAAAVAVPEHDGNNSNSSGMTMKGVRISGRPLYLDVQATSPVDPRVL :  74
Arabidopsis_thaliana : MASKVISATIRRTLTKPHGTFSRCRYLSTAAAATEVNYEDES-----IMMKGVRISGRPLYLDMQATTPIDPRVF :  70
Escherichia_coli     : -----------------------------------------------MMYGVYRAMKLPIYLDYSATTPVDPRVA :  28


Glycine_max_Chr01    : DAMLPFYLSR--YGNPHSRTHFYGWESDEAVEHARAQVASLIGASPKEIVFTSGATESNNISVKGVMHFYKDKKR : 147
Glycine_max_Chr11    : DAMLPFYLSR--YGNPHSRTHFYGWESDEAVEHARAQVASLIGASPKEIVFTSGATESNNISVKGVMHFYKDKKR : 147
Arabidopsis_thaliana : DAMNASQIHE--YGNPHSRTHLYGWEAENAVENARNQVAKLIEASPKEIVFVSGATEANNMAVKGVMHFYKDTKK : 143
Escherichia_coli     : EKMMQFMTMDGTFGNEASRSHRFGWQAEEAVDIARNQIADLVGADPREIVFTSGATESDNLAIKGAANFYQKKGK : 103

                                                               #   #                        #
Glycine_max_Chr01    : HVITTQTEHKCVLDSCRHLQQEGFDVTYLPVESDGLIDLDKLRAAIRPDTGLVSVMAVNNEIGVVQPMERIGRIC : 222
Glycine_max_Chr11    : HVITTQTEHKCVLDSCRHLQQEGFDVTYLPVESDGLIDLDELRASIRPDTGLVSVMAVNNEIGVVQPMEEIGRIC : 222
Arabidopsis_thaliana : HVITTQTEHKCVLDSCRHLQQEGFEVTYLPVKTDGLVDLEMLREAIRPDTGLVSIMAVNNEIGVVQPMEEIGMIC : 218
Escherichia_coli     : HIITSKTEHKAVLDTCRQLEREGFEVTYLAPQRNGIIDLKELEAAMRDDTILVSIMHVNNEIGVVQDIAAIGEMC : 178


Glycine_max_Chr01    : KEFNVPFHTDAAQALGKIPIDVEKWNVSLMSLSGHKIYGPKGVGALYMRRRPRIRVEPQMNGGGQERGIRSGTVP : 297
Glycine_max_Chr11    : KEFNVPFHTDAAQALGKIPIDVERWNVSLMSLSGHKIYGPKGVGALYMRRRPRIRVEPQMNGGGQERGIRSGTVP : 297
Arabidopsis_thaliana : KEHNVPFHTDAAQAIGKIPVDVKKWNVALMSMSAHKIYGPKGVGALYVRRRPRIRLEPLMNGGGQERGLRSGTGA : 293
Escherichia_coli     : RARGIIYHVDATQSVGKLPIDLSQLKVDLMSFSGHKIYGPKGIGALYVRRKPRVRIEAQMHGGGHERGMRSGTLP : 253
                                                         **

                               #
Glycine_max_Chr01    : TPLVVGMGSACEVAKNEMEYDEKRISALQQRLLNGIREKLDGVVVNGSLERRYVGNLNLSFAYVEGESLLMGLKE : 372
Glycine_max_Chr11    : TPLVVGMGAACEVAKDEMEYDEKRISALQQRLLNGIREKLDGVVVNGSLERRYVGNLNLSFAYVEGESLLMGLKE : 372
Arabidopsis_thaliana : TQQIVGFGAACELAMKEMEYDEKWIKGLQERLLNGVREKLDGVVVNGSMDSRYVGNLNLSFAYVEGESLLMGLKE : 368
Escherichia_coli     : VHQIVGMGEAYRIAKEEMATEMERLRGLRNRLWNGIKD-IEEVYLNGDLEHGAPNILNVSFNYVEGESLIMALKD : 327


Glycine_max_Chr01    : VAVSSGSACTSASLEPSYVLRALGVEEDMAHTSIRFGIGRFTTEAEIDRAVELTVQQVEKLREMSPLYEMVKEGI : 447
Glycine_max_Chr11    : VAVSSGSACTSASLEPSYVLRALGVEEDMAHTSIRFGIGRFTTEAEIDRAVELTVQQVEKLREMSPLYEMVKEGI : 447
Arabidopsis_thaliana : VAVSSGSACTSASLEPSYVLRALGVDEDMAHTSIRFGIGRFTTKEEIDKAVELTVKQVEKLREMSPLYEMVKEGI : 443
Escherichia_coli     : LAVSSGSACTSASLEPSYVLRALGLNDELAHSSIRFSLGRFTTEEEIDYTIELVRKSIGRLRDLSPLWEMYKQGV : 402
                               *

Glycine_max_Chr01    : NIKDIQWAQH : 457
Glycine_max_Chr11    : NIKDIQWAQH : 457
Arabidopsis_thaliana : DIKNIQWSQH : 453
Escherichia_coli     : DINSIEWAHH : 412
```
