# Supplementary material for: Differential expression of cysteine desulfurases in soybean
Source: BMC Plant Biol. 2011 Nov 18;11:166. doi: 10.1186/1471-2229-11-166 (PMC3233524; doi:10.1186/1471-2229-11-166)
Supplement: Additional file 2 — Alignment of SufS-like. Alignment of soybean cysteine desulfurase homologue to SufS from Escherichia coli. * indicates residues from active and from cofactor binding sites. # indicates amino acids residues that differ between soybean duplicated genes. [file 1471-2229-11-166-S2.PDF]

```
                                                                     #        ##
Glycine_max_Chr09    : MEVLLPKLPSFKFPSATYCCSITSRSSSYVRFGFRRVSVCASTVNETVAEPTVGSSSLGHSTRPHFPILHQEVNG :  75
Glycine_max_Chr15    : MEVLLPKLPSFKFPSATYCCSITSRSSSYVRFGFRRVSVCASTVNEAVAEPIVGFLSLGHSTRPHFPILHQEVNG :  75
Arabidopsis_thaliana : MEGVAMKLPSFPN-----AISIGHRSFSRVRCSSSLSVCSAAAASSATISTDSESVSLGHRVRKDFRILHQEVNG :  70
Escherichia_coli     : ------------------------------------------------------MTFSVDKVRADFPVLSREVNG :  21
Synechocystis_sp     : --MVALQIP-----------------------------------------------SLAATVRQDFPILNQEING :  26

                                       #
Glycine_max_Chr09    : SKLVYLDNAATSQKPTTVLKALQNYYEAYNSNVHRGIHFLSAKATDEYESARRKVASFINATDSREIIFTKNASE : 150
Glycine_max_Chr15    : SKLVYLDNAATSQKPTAVLKALQNYYEAYNSNVHRGIHFLSARATDEYESARRKVASFINAIDSREIVFTKNASE : 150
Arabidopsis_thaliana : SKLVYLDSAATSQKPAAVLDALQNYYEFYNSNVHRGIHYLSAKATDEFELARKKVARFINASDSREIVFTRNATE : 145
Escherichia_coli     : LPLAYLDSAASAQKPGQVIDAEAEFYRHGYAAVHRGIHTLSAQATEKMENVRKQASLFINARSAEELVFVRGTTE :  96
Synechocystis_sp     : HPLVYLDNAATSQKPRAVLEKLMHYYENDNANVHRGAHQLSVRATDAYEAVRNKVAKFINARSPREIVYTRNATE : 101

                                                                    #
Glycine_max_Chr09    : AINLVAYSWGLSNLKPDDEIILTVAEHHSAIVPWQIVAQKTGAVLNFVDLNQDEIPDIDKLKEMLSRKTKIVVVH : 225
Glycine_max_Chr15    : AINLVAYSWGLSNLKPDDEIILTVAEHHSAIVPWQIVAQKTGAVLKFVDLNQDEIPDIDKLKEMLSRKTKIVVVH : 225
Arabidopsis_thaliana : AINLVAYSWGLSNLKPGDEVILTVAEHHSCIVPWQIVSQKTGAVLKFVTLNEDEVPDINKLRELISPKTKLVAVH : 220
Escherichia_coli     : GINLVANSWGNSNVRAGDNIIISQMEHHANIVPWQMLCARVGAELRVIPLNPDGTLQLETLPTLFDEKTRLIAIT : 171
Synechocystis_sp     : AINLVAYSWGMNNLKAGDEIITTVMEHHSNLVPWQMVAAKTGAVLKFVQLDEQESFDLEHFKTLLSEKTKLVTVV : 176

                                   #
Glycine_max_Chr09    : HVSNVLASVLPIRDIAQWAHDVGAKVLVDACQSVPHMMVDVQSLNADFIVASSHKMCGPTGIGFLYGKIDLLSSM : 300
Glycine_max_Chr15    : HVSNVLASVLPIQDIAQWAHDVGAKVLVDACQSVPHMMIDVQSLNVDFIVASSHKMCGPTGIGFLYGKIDLLSSM : 300
Arabidopsis_thaliana : HVSNVLASSLPIEEIVVWAHDVGAKVLVDACQSVPHMVVDVQKLNADFLVASSHKMCGPTGIGFLYGKSDLLHSM : 295
Escherichia_coli     : HVSNVLGTENPLAEMITLAHQHGAKVLVDGAQAVMHHLVDVQALDCDFYVFSGHKLYGPTGIGILYVKEALLQEM : 246
Synechocystis_sp     : HISNTLGCVNPAEEIAQLAHQAGAKVLVDACQSAPHYPLDVQLIDCDWLVASGHKMCAPTGIGFLYGKEEILEAM : 251
                                                                            **

Glycine_max_Chr09    : PPFLGGGEMISDVYLDH-STYAEPPSRFEAGTPAIGEAIGLGAAIDYLSGIGMQTIHDYEVELGRYLYERLLSVP : 374
Glycine_max_Chr15    : PPFLGGGEMISDVYLDH-STYAEPPSRFEAGTPAIGEAIGLGAAIDYLSGIGMQTIHDYEVELGRYLYERLLSVP : 374
Arabidopsis_thaliana : PPFLGGGEMISDVFLDH-STYAEPPSRFEAGTPAIGEAIALGAAVDYLSGIGMPKIHEYEVEIGKYLYEKLSSLP : 369
Escherichia_coli     : PPWEGGGSMIATVSLSEGTTWTKAPWRFEAGTPNTGGIIGLGAALEYVSALGLNNIAEYEQNLMHYALSQLESVP : 321
Synechocystis_sp     : PPFFGGGEMIAEVFFDH-FTTGELPHKFEAGTPAIAEAIALGAAVDYLTDLGMENIHNYEVELTHYLWQGLGQIP : 325

Glycine_max_Chr09    : NIRIYGPAPSEKVERAALCSFNVENLHPTDLATLLDQQHGVAIRSGHHCAQPLHRYLGVSSSARASLYFYNTKED : 449
Glycine_max_Chr15    : NIRIYGPAPSEKVERAALCSFNVENLHPTDLATLLDQQHGVAIRSGHHCAQPLHRYLGVSSSARASLYFYNTKED : 449
Arabidopsis_thaliana : DVRIYGPRPSESVHRGALCSFNVEGLHPTDLATFLDQQHGVAIRSGHHCAQPLHRYLGVNASARASLYFYNTKDD : 444
Escherichia_coli     : DLTLYGPQN-----RLGVIAFNLGKHHAYDVGSFLDNY-GIAVRTGHHCAMPLMAYYNVPAMCRASLAMYNTHEE : 390
Synechocystis_sp     : QLRLYGPNP-KHGDRAALASFNVAGLHASDVATMVDQD-GIAIRSGHHCTQPLHRLFDASGSARASLYFYNTKEE : 398
                                                                       *

Glycine_max_Chr09    : VDNFIHALNDTVSFFN--SLM- : 468
Glycine_max_Chr15    : VDNFIHALNDTVSFFS--SLM- : 468
Arabidopsis_thaliana : VDAFIVALADTVSFFN--SFK- : 463
Escherichia_coli     : VDRLVTGLQRIHRLLG------ : 406
Synechocystis_sp     : IDLFLQSLQATIRFFSDDDFTV : 420
```
