# Supplementary material for: Differential expression of cysteine desulfurases in soybean
Source: BMC Plant Biol. 2011 Nov 18;11:166. doi: 10.1186/1471-2229-11-166 (PMC3233524; doi:10.1186/1471-2229-11-166)
Supplement: Additional file 3 — Alignment of ISD11 proteins. Alignment of soybean, Arabidopsis thaliana and Saccharomyces cerevisiae ISD11 proteins. [file 1471-2229-11-166-S3.PDF]

```
Glycine_max_Chr08         : MSAAASSSATPSAAQVLSLFRSLLRAAREFPDYNIREYTKRRTIDSFRHNATLSDPSQISTAFAHGKSQLAVVKR : 75
Glycine_max_Chr18         : MSALAASSATPSAAQALSLFRSLLRAAREFPDYNIREYTKLRTTDAFRHNATLSDPNSISTAFAHGKSQLAVVKR : 75
Arabidopsis_thaliana      : ---------MVSSSEVLSLCRALLRAGRQFPDYNIREYSKRRTLDGFRMNKNLTDPSKVTEAYAEAKKQLFVAER : 66
Saccharomyces_cerevisiae : ----MPGFTAPTRRQVLSLYKEFIKNANQFNNYNFREYFLSKTRTTFRKNMNQQDPKVLMNLFKEAKNDLGVLKR : 71


Glycine_max_Chr08         : QAVVYSLYDSPLRSVMELQQVPF : 98
Glycine_max_Chr18         : QAVVYSLYAPPLRNVMELQQTPF : 98
Arabidopsis_thaliana      : VLKVYLAYPPKTKNIMEVKLQ-- : 87
Saccharomyces_cerevisiae : QSVISQMYTFDRLVVEPLQGRKH : 94
```
