# Supplementary material for: Differential expression of cysteine desulfurases in soybean
Source: BMC Plant Biol. 2011 Nov 18;11:166. doi: 10.1186/1471-2229-11-166 (PMC3233524; doi:10.1186/1471-2229-11-166)
Supplement: Additional file 4 — Primer sequences. Primer sequences and amplicon characteristics for each gene. [file 1471-2229-11-166-S4.PDF]

Additional file 4 Primer sequences and amplicon characteristics for each gene.

Gene	Forward primer sequence (5'-3')	Reverse primer sequence (5'-3')	Amplicon size (bp)	Locus[a]
NFS1_Chr01	CGGAGCACAAGTGCGTCC	CCTCCCAATTCTCTCCATCGGT	200	Glyma01g40510
NFS1_Chr11	CGGAGCACAAGTGCGTCC	CCTCCCAATTTCCTCCATGGGC	200	Glyma11g04800
NFS2_Chr09	GTCGAACGAGCTGCCCTTTG	CCCGTGCACTTGAGCTGACA	157	Glyma09g02450
NFS2_Chr15	GTCGAACGAGCTGCCCTTTG	CACGTGCACTTGAGCTGACG	157	Glyma15g13350
F-BOX	AGATAGGGAAATGTTGCAGGT	CTAATGGCAATTGCAGCTCTC	93	CD397253
Metallop.	ATGAATGACGGTTCCCATGTA	GGCATTAAGGCAGCTCACTCT	114	AW310136

[a] Phytozome or NCBI database accession number.
